# A geostatistical approach to estimating source apportionment in urban and peri-urban soils using the Czech Republic as an example

**DOI:** 10.1038/s41598-021-02968-8

**Published:** 2021-12-08

**Authors:** Prince Chapman Agyeman, Kingsley JOHN, Ndiye Michael Kebonye, Luboš Borůvka, Radim Vašát, Ondřej Drábek

**Affiliations:** Department of Soil Science and Soil Protection, Faculty of Agrobiology, Food and Natural Resources, Czech University of Life Sciences Prague, 16500 Prague, Czech Republic

**Keywords:** Environmental chemistry, Environmental impact, Risk factors

## Abstract

Unhealthy soils in peri-urban and urban areas expose individuals to potentially toxic elements (PTEs), which have a significant influence on the health of children and adults. Hundred and fifteen (n = 115) soil samples were collected from the district of Frydek Mistek at a depth of 0–20 cm and measured for PTEs content using Inductively coupled plasma—optical emission spectroscopy. The Pearson correlation matrix of the eleven relevant cross-correlations suggested that the interaction between the metal(loids) ranged from moderate (0.541) correlation to high correlation (0.91). PTEs sources were calculated using parent receptor model positive matrix factorization (PMF) and hybridized geostatistical based receptor model such as ordinary kriging-positive matrix factorization (OK-PMF) and empirical Bayesian kriging-positive matrix factorization (EBK-PMF). Based on the source apportionment, geogenic, vehicular traffic, phosphate fertilizer, steel industry, atmospheric deposits, metal works, and waste disposal are the primary sources that contribute to soil pollution in peri-urban and urban areas. The receptor models employed in the study complemented each other. Comparatively, OK-PMF identified more PTEs in the factor loadings than EBK-PMF and PMF. The receptor models performance via support vector machine regression (SVMR) and multiple linear regression (MLR) using root mean square error (RMSE), R square (R^2^) and mean square error (MAE) suggested that EBK-PMF was optimal. The hybridized receptor model increased prediction efficiency and reduced error significantly. EBK-PMF is a robust receptor model that can assess environmental risks and controls to mitigate ecological performance.

## Introduction

Human-related activities such as industry, sewage discharge, mining, atmospheric deposition, and agriculture are primarily characterized by urban and peri-urban soil^1^. International communities, allied bodies, multinational companies, countries, and humans who are directly affected by potentially toxic elements (PTEs) worldwide have expressed great concern about the threat posed by PTEs. PTEs accumulations in the soil can cause changes in soil fertility and cultivation characteristics of bioavailability, as well as increase the persistence of PTEs toxicity, which can easily be transported and accumulated in a food chain, resulting in food safety hazards and health-related issues in the human body via a variety of pathways (inhalation, ingestion, and dermal uptake)^2–4^. Public quibble about the build-up of PTEs in farmland has been escalating, limiting the soil's functionality, creating crop and water toxicity, and endanger human health^5,6^. The impact of PTEs on the soil is a cross-border challenge that is not limited to a particular region but also a worldwide concern, which transcends peri-urban, urban and continental borders. Global integration, trade and movement of goods and services facilitate the impact of PTEs from afar on someone distant from a polluted place. Urban and rural areas, according to Kombe^7^ and Keshavarzi et al.^8^, are transitional areas where activities are integrated. This allows for easy accessibility of goods and services and migration of PTEs through torrential rainfall and erosion from urban and peri-urban areas and contrariwise. However, some big cities are expanding in order to incorporate a rising population in peri-urban areas^8^ closer to urban areas. Though some cities are closer to peri-urban areas, it allows for easy congestion in the towns due to it being a hub for most multinational industries and people having the edge of migrating urban, increases vehicular traffic, creates an avenue for urban expansion and construction activities that contribute to soil pollution in the immediate environment.

According to Vázquez Cueva et al.^9^ and Tume et al.^10^, in many instances, urban waste, industrial effluents, and even manures and agricultural fertilizers pollute the soils of these locations with PTEs. Anthropogenic pollutants such as leaks and spills, manufacturing and construction activities, agricultural practices, transportation and chemical waste dumping, concomitant with natural pollutants, predominate in urban areas, gradually drifting to the peri-urban area as a result of land acquisition, industrial and urban expansion.

The uniqueness and dynamism of each urban and peri-urban area differ from one another geographically. However, the only constant is that PTEs are resident in the soil due to pollution, whether anthropogenic, natural, or both. Fei et al.^11^ and Huang et al.^12^ outlined that to minimize the cost and complexity of soil remediation effectively, it is critical to quantify the sources of soil PTEs pollution. The practicality of evidence-based analysis can be relished based on the robustness of the statistical approaches employed either qualitatively or quantitatively. Source apportionment approaches have been applied in multiple disciplines, including soil science, water research, and air quality assessment. Positive matrix factorization (PMF), absolute principal components score-multiple linear regression (APCS-MLR), UNMIX, and chemical mass balance (CMB) are some of the multivariate statistics utilized in the quantification of source apportionment of pollutants. However, authors frequently apply the PMF, and APCS-MLR approaches to quantify source distribution. Lang et al.^13^; Jain et al.^14^; Guan et al.^15^; Salim et al.^16^; Zhang et al.^17^; Fei et al.^4^; Zhang et al.^18^ and Agyeman et al.^19^, are some of these authors that fall on the resilience of PMF and APCS-MLR to calculate source apportionment. The healthy academic nemesis between PMF and APCS-MLR has complemented each other in academic space. However, because the terrain (soil science) is so important, most authors sought to apply either one or both in source apportionment. Most comparative analyses, to name a few, Gholizadeh et al.^20^, Salim et al.^16^; Jain et al.^14^ and Guan et al.^15^ have adjudged PMF or APCS/MLR to be optimal. As summarized by Lee et al.^21^, the preference for PMF or APCS/MLR or both over the other receptor models based on the competitive advantage such as (i) the use of efficient monitoring processes, the establishment of a sizeable database which has become a general practice;(ii) these receptor models do not require pre-measured source profiles (i.e., backward tracking) in discrepancy with chemical mass balance (CMB); and (iii) the receptor model's capability permits it to cope with significant amounts of monitoring data. However, if the applicability of PMF or APCS/MLR or both has an advantage over other receptor models, its excellent performance is hampered by various limitations or constraints. According to Yuanan et al.^22^, PMF may produce inaccurate estimations if the PTEs identified in topsoil have undergone significant selection enrichment. Furthermore, Wu et al.^23^ and Guan et al.^15^ claimed that PMF was unable to effectively determine the nature of the differences in PTEs observed in surface soils across the entire area and create a fitting effect. Zhang et al.^17^ also added that APCS/MLR could not discharge a lot of sources in each factor loadings.

Investigating pollution sources pathways via diverse receptor models aids in controlling pollution hazards in the environment. The use of robust receptor models facilitates in minimizing the risk of pollution and, at the same time, can assist in assuaging occurrences. Essentially, the pathways of pollution sources may be identified using receptor models. The output obtained assists stakeholders in evaluating health and ecological impact and adopting actions to improve sustainability impact. The development of robust receptor models aids in detecting locations that require further attention and assists stakeholders in developing reliable emergency response plans. Wang et al.^24^ stressed that applying receptor models, which are based on multivariate statistical approaches to identify and quantify pollutants (PTEs) apportionment to their sources, can significantly improve the traditional source apportionment approach. This study intends to use PMF as a base model to build a hybridized receptor model that will enhance efficiency and minimize errors in identifying and estimating source apportionment. PMF will be combined with geostatistical approaches such as ordinary kriging and empirical Bayesian kriging. The study region is an active agricultural area with many industries such as metal works and steel industries. We hypothesized that the dependability of the receptor model is determined by its efficiency and ability to reduce error when applied. This study addresses the following research question: How reliable are the hybridized receptor models compared to the base model (PMF)? What is the performance of the receptor models in terms of efficiency and error reduction? The specific objectives of this paper revolve around the following: determining the concentration of PTEs in urban and peri-urban soil, comparing diverse receptor models for source apportionment, and proposing and validating receptor model technique that is efficient and more practical for source apportionment estimation.

## Materials and methods

### Research location (case study)

**Figure 1:**
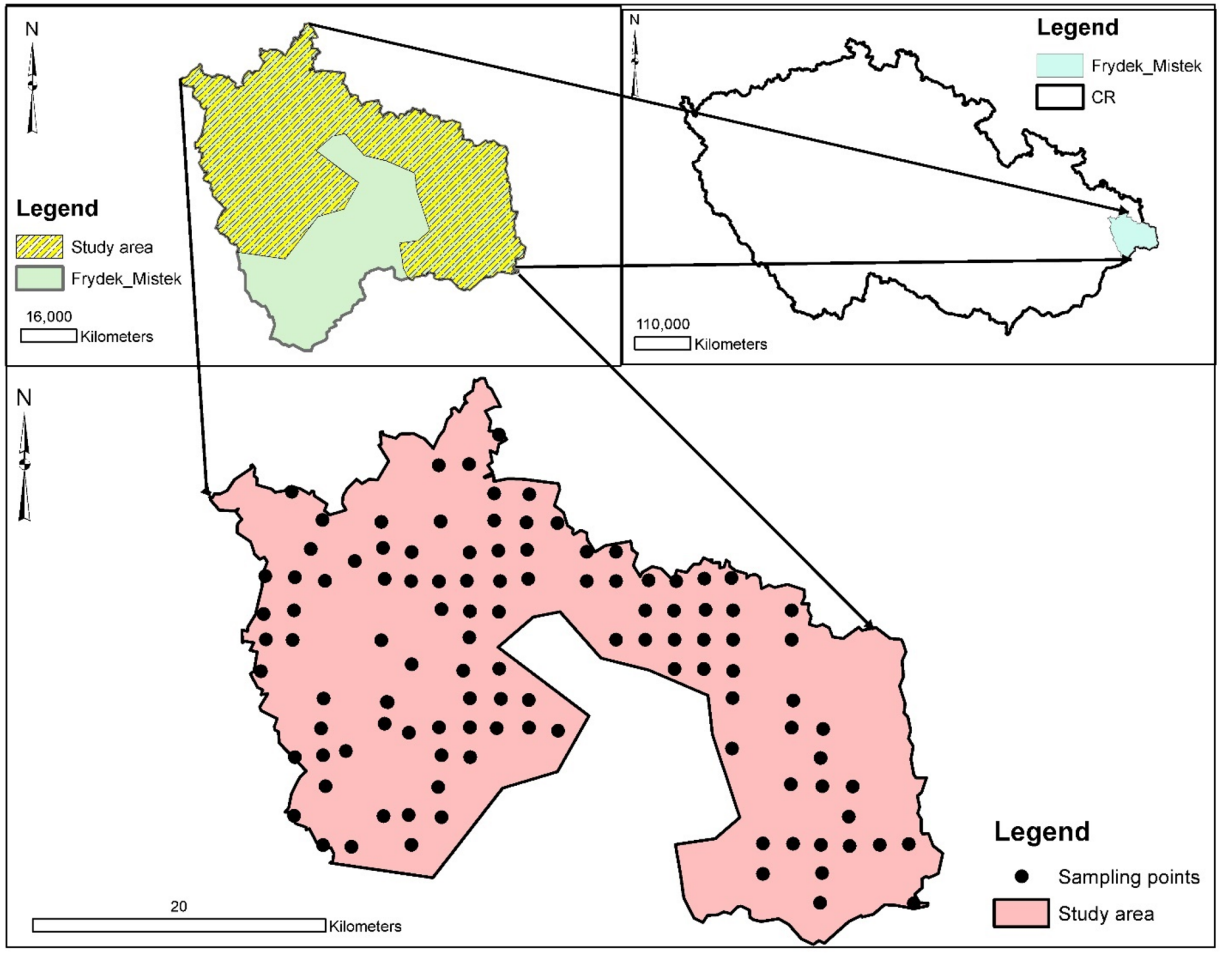
Study area.

The selected study area is in the Czech Republic in the Frydek Mistek district in the Moravian-Silesian area (Fig. 1). The research area's geomorphology is relatively rugged terrain, mostly part of the Moravian-Silesian Beskydy region, a part of the extracellular matrix mountain range. The study area is positioned at latitude 49° 41′ 0′ North and longitude 18° 20′ 0′ East at an altitude ranging from 225 to 327 m above sea level; however, the Koppen classification system of the area's climatic condition is classified as Cfb = temperate oceanic climate with a high level of rainfall even in dry months. The temperature fluctuates typically from − 5 to 24 °C throughout the year, with temperatures occasionally falling below − 14 °C or reaching over 30 °C. The maximum average annual rainfall is 83 mm, with a minimum total accumulation of 17 mm^25^. The district's area survey is estimated to be 1208 km^2^, with 39.38% of the land area under cultivation and 49.36% under forest cover. However, the site designated for the study is approximately 889.8 km^2^ (see Fig. 1). Agriculture, the steel industry, and metal works are all active in and around the Ostrava neighborhood. The soil qualities are easily distinguished from the color, texture, and carbonate concentration of the soil. The soil's texture is medium to fine, and it is derived from parent materials. They are primarily colluvial, alluvial, or aeolian in nature. Some soil areas have mottles in the top and subsoil, which are usually followed by concrete and bleaching. However, cambisols and stagnosols are the most common soil types in the region^26^. With elevations ranging from 455.1 to 493.5 m, cambisol soils predominate in the Czech Republic^27^.

### Soil sampling and soil analysis

One hundred and fifteen topsoil samples were collected from urban and peri-urban areas in the Frydek Mistek district. The sample design used was the regular grid, and the soil sample intervals were 2 × 2 km using a portable GPS unit (Leica Zeno 5 GPS) at a depth of 0 to 20 cm for topsoil. The samples were put in Ziploc bags, labelled correctly, and brought to the laboratory. To obtain a pulverized sample, the samples were air-dried, crushed by a mechanical device (Fritsch disk mill), and sieved (< 2 mm). One gram of the dried, homogenized, and sieved soil sample (sieve size 2 mm) was placed in a labelled Teflon bottle. In each Teflon bottle, 7 ml of 35% HCl and 3 ml of 65% HNO_3_ were dispensed (using automatic dispensers—one for each acid). The cap was gently closed to allow the sample to remain overnight for reaction (aqua regia procedure). Subsequently, the supernatant was placed on a hot metal plate for 2hrs to boost the digestion process of the sample before being allowed to cool. Then, the supernatant was transferred to a 50 ml volumetric flask and diluted to 50 ml with deionized water. After that, the diluted supernatant was filtered into 50 ml PVC tubes.

Furthermore, 1 ml of the diluted solution was diluted with 9 ml of deionized water and filtered into a 12 ml test tube prepared for PTE (Al. Ba, Cd, Pb, Sb, Fe. V) pseudo-concentration. ICP-OES (inductively coupled plasma optical emission spectrometry) (Thermo Fisher Scientific, USA) was used to detect metal concentrations in accordance with conventional methods and protocols. The quality assurance and control (QA/QC) method was ensured by examining each sample's standards reference material (SRM NIST 2711a Montana II soil). The detection limits of the PTEs used in this investigation are as follows: 0.0002 (Cd), 0.0007 (Cr), 0.0060 (Cu), 0.0001 (Mn), 0.0004 (Ni), 0.0015 (Pb), 0.0067 (As), and 0.0060. (Zn). To accomplish QA/QC, we used blank reagents, repeated samples, and standard reference materials. Duplicate analysis was performed to guarantee that the error was minimized (< 5%).

### Receptor models

#### PMF receptor model

Positive matrix factorization (PMF) receptor modelling is often performed with the US-EPA PMF 5.0 software^28^. The receptor model is one of the multivariate approaches for source analysis used to solve the chemical mass balance, and the original data matrix X is represented in the order m × n, which can be written as

1 $$X = GF + E$$

G (m × p) represents a factor contribution matrix, F (p × n) also denotes the factor profile matrix, and E (m × n) is a residual error matrix. *E* is given as

2 $$e_{ij} = \mathop \sum \limits_{k = 1}^{p} g_{ik } f_{ki} - x_{ij}$$

where *i* is the elements 1 to *m*, *j* signifies elements 1 to *n*, and *k* represents the source from 1 to *p*. The authors have previously discussed the function of the minimal Q and the uncertainty, and the parameters and implementation techniques involved^19^.

#### Ordinary kriging - positive matrix factorization (OK-PMF)

Ordinary kriging (OK) is an interpolation approach that allowed us to estimate the spatial distribution of PTEs in the site under investigation. Kriging is an interpolation that predicts variable values in areas where data are unavailable based on the spatial pattern of the existing data^29^. The equation is expressed as

3 $${\text{\rm Z}}^{{\prime }} (x_{0} ) = \mathop \sum \limits_{i = 1}^{n} \lambda_{i} \cdot {\text{\rm Z}}(x_{i} )$$

It can be computed by the semi-variance function of the variables on the condition that the estimated value is unbiased and optimal. The semivariogram model is expressed as:

4 $$\gamma \left( h \right) = \frac{1}{2N\left( h \right)}\mathop \sum \limits_{i = 1}^{n} [Z(X_{i} ) - Z\left( {X_{i} + h} \right)]^{2}$$

whereby γ(h) signifies semi-variance, N(h) denotes point group number at distance h, Z(x_i_) represents numerical value at position x_i_, and Z (x_i_ + h) is the numerical value at a distance (x_i_ + h).

However, the hybridized (OK-PMF) equation between PMF and OK is given as

5 $${\text{\rm Z}}^{{\prime }} (x_{0} )_{ij} = \mathop \sum \limits_{i = 1}^{n} \lambda_{i} \cdot {\text{\rm Z}}(x_{i} )_{ij}$$

In which Z′$(x_{0} )_{ij}$ is the interpolated value for point $x_{0}$ of each PTEs from the kth source in the ith sampling location, $Z(x_{i} )_{ij}$ denotes a known value of the concentration of the single PTE in the soil in the jth source from the ith sampling site, and λ_i_ represent the kriging weight for the ${\text{Z}}(x_{{\text{i}}} )$ values.

The OK-PMF receptor model application is based on interpolated data points from all PTE data points observed. Predicted data from OK interpolation is retrieved and then fed into the US-EPA PMF 5.0 software for source contribution computation to estimate the PTE source distribution. The traditional PMF method uses raw data, but the OK-PMF approach uses predicted data after OK interpolation.

#### Empirical Bayesian kriging-positive matrix factorisation (EBK-PMF)

Empirical Bayesian kriging (EBK) is one of the numerous geostatistical interpolation techniques used in modelling in diverse fields such as soil science. Unlike the other kriging interpolation techniques, EBK varies from conventional kriging methods by considering the error of the semi variogram model estimation^30^. In EBK interpolation, several semi variogram models are calculated during the interpolation instead of a unitary semi variogram. The interpolation technique makes way for associated uncertainties, thereby plotting semi variogram and programming the highly complex parts to compose a good kriging approach^31^. The interpolation process of EBK follows three criteria as proposed by Krivoruchko^30^, (a) the model estimate semi variogram from the input dataset (b) based on the generated semi variogram a new predicted value is assigned to each inputted dataset location and (c) finally a model is computed from the simulated dataset. The Bayesian equation rule is giving as posterior

6 $$Prob\left( {A,B} \right) = \sum Prob \left( A \right) \times \frac{Prob(B/A)}{{Prob\left( B \right)}}$$

The semi variogram calculation is based on the Bayes rule, which indicates that the semi variogram may generate the observed dataset. Krivoruchko^30^ explains that, during the computation of semivariogram in step 1, a set of data is utilized to stimulate a new location input; however, steps 2 and 3 are replicated.

Nonetheless, the hybridized (EBK-PMF) equation between PMF and EBK is given as

7 $$Prob\left( {A,B} \right)_{ij} = \sum Prob \left( A \right) \times \frac{{Prob(B/A)_{ij} }}{{Prob(B)_{i} }}$$

where the $Prob\left( {A,B} \right)_{ij}$ represents the posterior probability of the computed PTEs from the kth source in the ith sampling location, $Prob\left( A \right)$ represent the prior probability, $Prob (B,A)_{ij}$ denotes the likelihood of the concentration of the single PTE in the soil in the jth source from the ith sampling site and the $Prob (B)_{i}$ also signifies the marginal probability. The EBK-PMF receptor model application is based on interpolated data points from all observed PTE data points. To estimate the PTE source distribution, predicted data from EBK interpolation is retrieved and then inserted into the US-EPA PMF 5.0 software for source contribution computation. The traditional PMF approach uses raw data, but the hybridized EBK-PMF uses predicted data after EBK interpolation.

#### Geographically weighted ordinary regression (GWR-OLS)

Geographically weighted regression (GWR) advances the well-known regression architecture by predicting a set of parameters for any range of locations throughout a study region instead of a single collection of parameters. Four environmental covariates (i.e., elevation, total catchment area, LS factor and valley depth) were extracted and used to fit a GWR-OLS model to predict the distribution of PTEs in each factor loading based on the factors scores obtained from each receptor model. In the first place, each equation (elevation, total catchment area, LS factor, and valley depth) was optimized using the GWR.sel function from the spgwr R package. Subsequently, the GWR function was applied to fit the GWR-OLS depending on the bandwidth determined by the previous function. Brunsdon et al.^32^ provide detailed descriptions of the GWR-OLS. Zhang et al.^33^ ; Kumar et al.^34^ ; Wang et al.^35^ ; Song et al.^36^ ; Zeng et al.^37^ and Wang et al.^38^ are only a few of the soil-based research that has effectively used the GWR-OLS for various reasons. The predicted factor scores data from GWR-OLS were then kriged to generate a geographical weighted regression kriging spatial distribution map for the factor scores of each receptor model.

### Data modelling techniques

#### Support vector machine regression (SVMR)

SVM is a machine learning algorithm that develops an optimal disengaging hyperplane to separate categories with similarities but is not linearly independent. Vapnik^39^, created the technique for classification reasons; however, it has recently been used to solve regression-oriented problems. According to Li et al.^40^, SVM is one of the best classifier approaches and has been used in a variety of fields. The regression aspect of SVM is used in this study (support vector machine regression-SVMR). Cherkassky and Mulier^41^, pioneered SVMR as a regression based on a kernel, and its computation was performed using a linear regression model with a multinational space feature. However, according to John et al.^42^ the SVMR modelling employs a hyperplane linear regression, which generates a nonlinear relationship and allows for the space feature. Vohland et al.^43^, suggested that epsilon (ε)-SVMR uses a trained dataset to obtain a represented model as an epsilon -insensitive feature utilized to map data independently with the optimum epsilon- ε departure from dependent data training.

The preset distance error inside is ignored from the actual value, and if the error is larger than the epsilon(ε), the soil attribute compensates for it. In addition, the model decreases the complexity of training data to a broader subset of support vectors. The equation as proposed by Vapnik^39^, is given as

8 $$y\left( x \right) = \mathop \sum \limits_{k = 1}^{N} \alpha_{k} K\left( {x, x_{k} } \right) + b,$$

In which the b represents the scalar threshold, $K\left( {x ,x_{k} } \right)$ representing the kernel function, $\alpha$ denoting the Lagrange multiplier, N symbolizing the number dataset, $x_{k}$ representing the data input, and $y$ is the data output. One of the critical kernels used is the SVMR operation with the Gaussian Radial Basis Function (RBF). The RBF kernel was applied to ascertain the optimum SVMR model that is essential to procure the finest penalty set factors C and the kernel parameters gamma (γ) for the PTEs training data. We assessed the set of training and then tested the validation set's model performance. The application of SVMR is simple. When compared to other regression approaches, SVMR requires less computing. Furthermore, SVMR employs multiple classifiers that have been trained on various types of data using probability principles.

#### Multiple linear regression

The multiple linear regression (MLR) model is a regression model that encapsulates the relationship between a response variable and numerous predictor variables by employing linearly inserted parameters that are computed using the least-squares approach. In MLR, the least square model is a prediction function that is directed toward a soil attribute following the selection of an explanatory variable. The PTEs was used as the response variables, which was used to establish the linear relationship utilizing the explanatory variable. The MLR equation is given as

9 $$y = a + \mathop \sum \limits_{i - 1}^{n} b_{1 } {\text{{\rm X} }} x_{i} \pm \varepsilon_{i}$$

In which y represents the response variable, a denotes the intercept, n signifies the number of predictors, ${b}_{1}$ denotes the partial regression of coefficient, ${x}_{i}$ implies the predictors or the explanatory variables and the ${\varepsilon }_{i}$ signifies the error in the model, which is also called residual.

The model was utilized in R (K = 10 folds cross-validation, which is repeated five times). MLR can calculate the relative relevance of one or multiple predictor differences in proportion to the significance value. MLR refers to the ability to identify outliers or irregularities.

#### Data partitioning

The number of samples used in the modelling approaches was 115, and a random approach was used to divide the data into a test dataset (with 25% for validation) and a training dataset (75% for calibration). The training dataset was used to calibrate the regression models, while the test dataset was utilized to assess generalization capabilities^44^. This was done to evaluate the suitability of the various models used to estimate PTE source apportionment. All the models were put through a 10-fold cross-validation process and it was repeated five times. To predict the targeted variables, the factor contributions for each receptor model were employed as predictors or explanatory variables. All the modelling regimes were performed in a RStudio.

#### Accuracy assessment and validation

While evaluating the model's accuracy and its validation, validation criteria were used to establish the best and most optimal model fitting for the computation of source distribution based on geostatistical assessment-based positive matrix factorization receptor models. The receptor models were assessed utilizing mean absolute error (MAE), root mean square error (RMSE), and R square, or coefficient determination (R^2^). R^2^ illustrates the variation of the percentage in the response and is expressed by the regression model. The RMSE and the size of the variability within the independent measurement characterize the model prediction capability, while MAE establishes the true measurable value. The R^2^ value ought to be high to establish the optimum receptor model using the validation criteria, and the closer the value is to 1, the higher the accuracy. Corresponding to Li et al.^45^, R^2^ criteria value of 0.75 or less is considered a satisfactory prediction and above 0.75 is a good prediction. Methods for assessing validation requirements utilizing RMSE and MAE, with a lower obtained value being appropriate and ideal for model selection. The following equation describes the validation procedures.

#### Mean absolute error

10 $$MAE = \frac{1}{n}\mathop \sum \limits_{i = 1}^{n} Y_{i} - \hat{Y}_{i}$$

#### R square

11 $${\text{R}}^{2}\, \left( {{\% }} \right) = 1 - \frac{{\sum ({\text{Y}}_{{\text{i}}} - {\hat{\text {Y}}}_{{\text{i}}} )^{2} }}{{\sum ({\text{Y}}_{{\text{i}}} - {\hat{\text{Y}}}_{{\text{i}}} )^{2} }}$$

#### Root mean square error

12 $$RMSE\,\left( mg/kg \right) =\sqrt{\frac{1}{n}\mathop \sum \limits_{i = 1}^{n} (\hat{Y}_{i} - Y_{i} )^{2}}$$

whereby n represents the size of the observations $Y_{i}$ represents the measured response and the $\hat{Y}_{i}$ also stated as the predicted response values, accordingly, for the ith observation term.

### Data analysis

The R studio was used to perform correlation matrix, support vector machine regression, multiple linear regression and the geographically weighted ordinary regression. Ordinary kriging and empirical Bayesian kriging were interpolated in ArcGIS.

## Results and discussion

### Data description

The statistical description of the geometric mean concentration of the PTEs in the study area is shown in Table 1. According to the estimated coefficients of variation (CV) of the PTEs, the CV of Ba, Cd, and Pb surpassed 50% (see Table 1), implying that the sampled data are highly variable and non-homogeneous pollution caused by anthropogenic activities. In contrast, the CV of the following PTEs Al, Fe, Sb, and V was less than 50%, indicating moderate variability and implying that the PTEs data is more homogeneously distributed. The standard deviation (SD) values obtained for each PTE exceeded one. They were relatively high due to the high values of some of the PTEs, implying that the PTEs are highly variable. The minimum and maximum values of the PTEs ranges between 6284.59 and 27,709.33 mg/kg (Al), 29.80–265.66 mg/kg (Ba), 8650.32–79,901.24 mg/kg (Fe), 2.26–9.72 mg/kg (Sb), 15.61–81.86 mg/kg (V), 0.61–7.28 mg/kg (Cd) and 9.56 mg/kg to 155.69 mg/kg (Pb). The minimum and maximum values for the environmental covariates were 240.33–902.11 for elevation, 984.56–12,617,766.68 for total catchment area, 0.01–13.08 for LS factor, and valley depth 25.73–351.13. The geometric mean concentration of Sb, Cd and Pb were found to be higher than the geochemical background level of both the world average values (WAV) and the European average values (EAV). The current study's antimony (Sb), cadmium (Cd), and lead (Pb) concentration levels were found to be 3.89, 13.14, and 1.25 times higher than the WAV threshold, and 2.50, 6.57, and 1.05 times higher than the EAV threshold, respectively. However, the geometric mean concentration of barium (Ba) and vanadium was below the geochemical threshold level of both WAV and EAV. The geometric mean of Cd in the current study was found to be higher when compared to the peri-urban soil of southeast China^1,46^. The geometric mean concentration of Fe, Pb, and Sb reported by Hossain Bhuiyan et al.^47^ (Dhaka [Fe 12,232 mg/kg]), Linde et al.^48^ (Sweden[Pb— 30 mg/kg]) , Tume et al.^49^ (Chile[Pb—19.8 mg/kg]),Wiseman et al.^50^ (University of Toronto Canada[Sb—0.68 kg/mg]) and De Miguel et al.^51^ (Madrid[Sb—1.01 mg/kg]) were found to be lower than the geometric mean concentrations of Fe, Pb, and Sb in the current study. Nadal et al.^52^ reported a low vanadium concentration of 19.3 mg/kg in an industrial area and 13.6 mg/kg in the residential area of Tarragona County, Spain, which was lower than the V concentration in the current study. Da Silva et al.^53^ reported low Ba concentration measured in five cities in Florida State (USA) such as Clay County (23.4 mg/kg), Orlando (20.3 mg/kg), Pensacola (48.1 mg/kg), Tampa (23.7 mg/kg) and West Palm Beach (29.1 mg/kg). In Thonburi in Bangkok, the geometric mean of Al measured in the urban soil was 13,800 mg/kg^54^, which was a bit higher than the mean concentration of Al measured in the current study. This implies that Thonburi, Bangkok, is inundated with more industrial activities that pollute the urban soil than the current study area.

**Table 1:** Statistical summary of sampled data.

Elements	Mean	S.D.*	Coef. Var.^$^	Minimum value	Maximum value	WAV^#^	EAV^@^
	mg/kg						
Al	13,251.08	3485.04	26.3	6284.59	27,709.33	–	–
Ba	79.46	40.83	51.38	29.8	265.66	460	400
Fe	20,054.95	9942.49	49.58	8650.32	79,901.24	–	–
Sb	2.61	1.08	41.33	2.26	9.72	0.67	1.04
V	31.37	9.35	29.81	15.61	81.86	129	68
Cd	1.84	1.01	55.14	0.61	7.28	0.14	0.28
Pb	33.86	18.51	54.68	9.56	155.69	27	32
**Environmental covariates**							
Elevation	378.36	93.55	24.72	240.33	902.11	–	–
Total Catchment Area	335,276.61	1,512,375.8	451.08	984.56	12,617,766.68	–	–
LS-Factor	1.29	1.66	129.06	0.01	13.08	–	–
Valley Depth	220.12	57.74	26.23	25.73	351.13	–	–

*Standard deviation, ^$^coefficient of variability, ^#^world average value and ^@^European average value^85^.

### Pearson correlation matrix (PCM) of the PTEs

**Figure 2:**
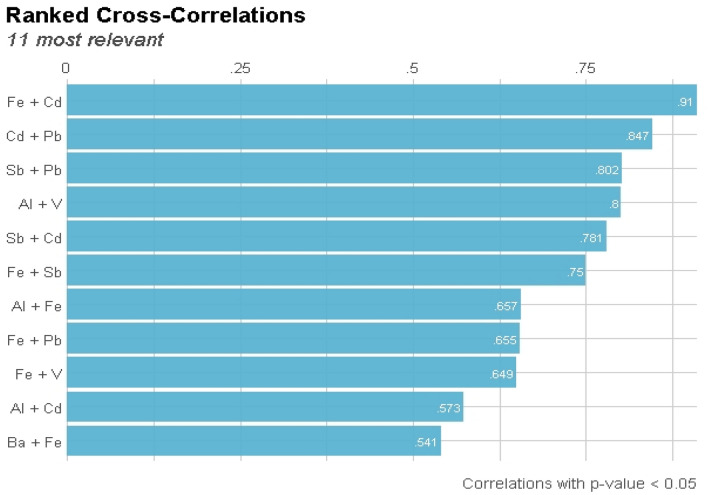
Interaction of PTEs using Pearson correlation matrix.

The metallic association among PTEs was identified using PCM to navigate metadata on the metallic pathways of the elements via their sources (see Fig. 2). The computed PCM revealed eleven optimal associations between the PTEs from moderate to high metallic strength. Cadmium exhibited a high correlation with Fe, Pb, and Sb, with r values of 0.91,0.847 and 0.781. The significant metallic nature of Cd and Pb (r = 0.847) reflects a geochemical tendency that is most likely related to the use of fertilizers and pesticides. This is congruent with the findings of Zhang et al.^55^ who reported that pesticides and fertilizers are most likely input sources for the Cd and Pb relationship. Cadmium and iron are industrially related due to steel and iron industries as well as non-ferrous metal production. According to Ursnyová and Hladková^56^, the emission of Cd to the atmosphere that precipitates on the soil surface is mostly caused by the steel, iron, and non-ferrous metal industries. However, Sb showed strong nexus with Pb and Cd with r value = 0.802 and 0.781, respectively. These PTEs (Sb, Cd, and Pb) have a close relationship in the battery manufacturing industry^57^. Aluminum (Al) and Vanadium (V) are also strongly associated with r value = 0.80. Al and V share the same source, according to Negri et al.^58^ and Harford et al.^59^, which is wastewater discharged from alumina refineries. Nevertheless, other PTEs such as FeV, FeBa, FePb, AlFe and AlCd also exhibited moderate relationship amongst each other with r values = 0.649, 0.541, 0.655, 0.657 and 0.573 respectively. Sedimentary ironstone that is rich in Fe oxide and contained a considerable amount of an iron ore compound from which iron (Fe) may be smelted economically and is defined to contain a large amount of Fe oxides is frequently deposited with Pb, V, and Ba in high concentration^60^. Based on their correlation, the correlation between Al and Fe is lithogenic, but the relationship between Al and Cd is more of a crustal origin^61^.

### Source identification and contribution

**Figure 3:**
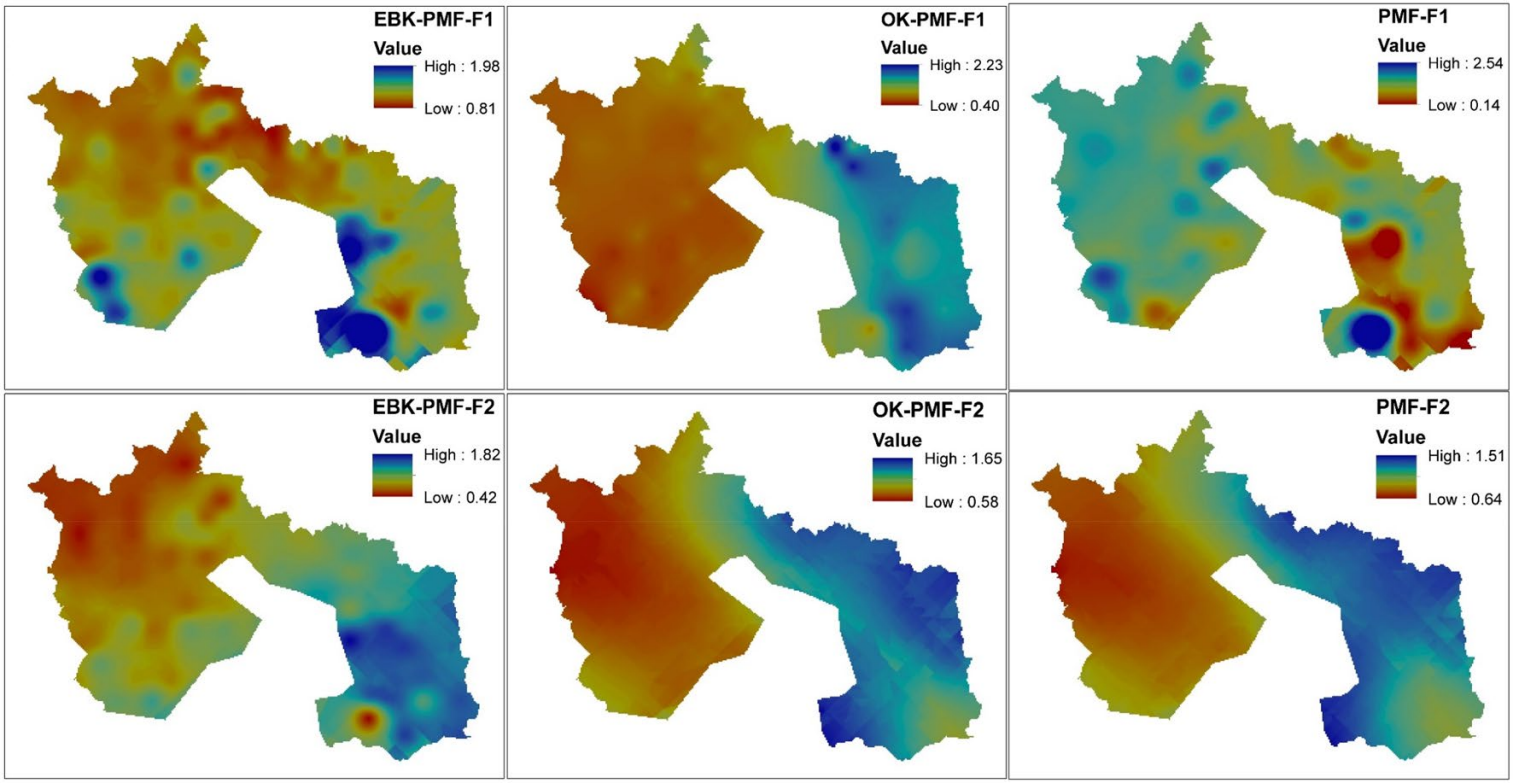
Spatial prediction of receptor model factor scores using geographically weighted regression kriging [Created in ArcGIS version 10.7 [The spatial distribution maps was created with ArcGIS Desktop (ESRI, Inc, Version 10.7, URL: https://desktop.arcgis.com)].

**Figure 4:**
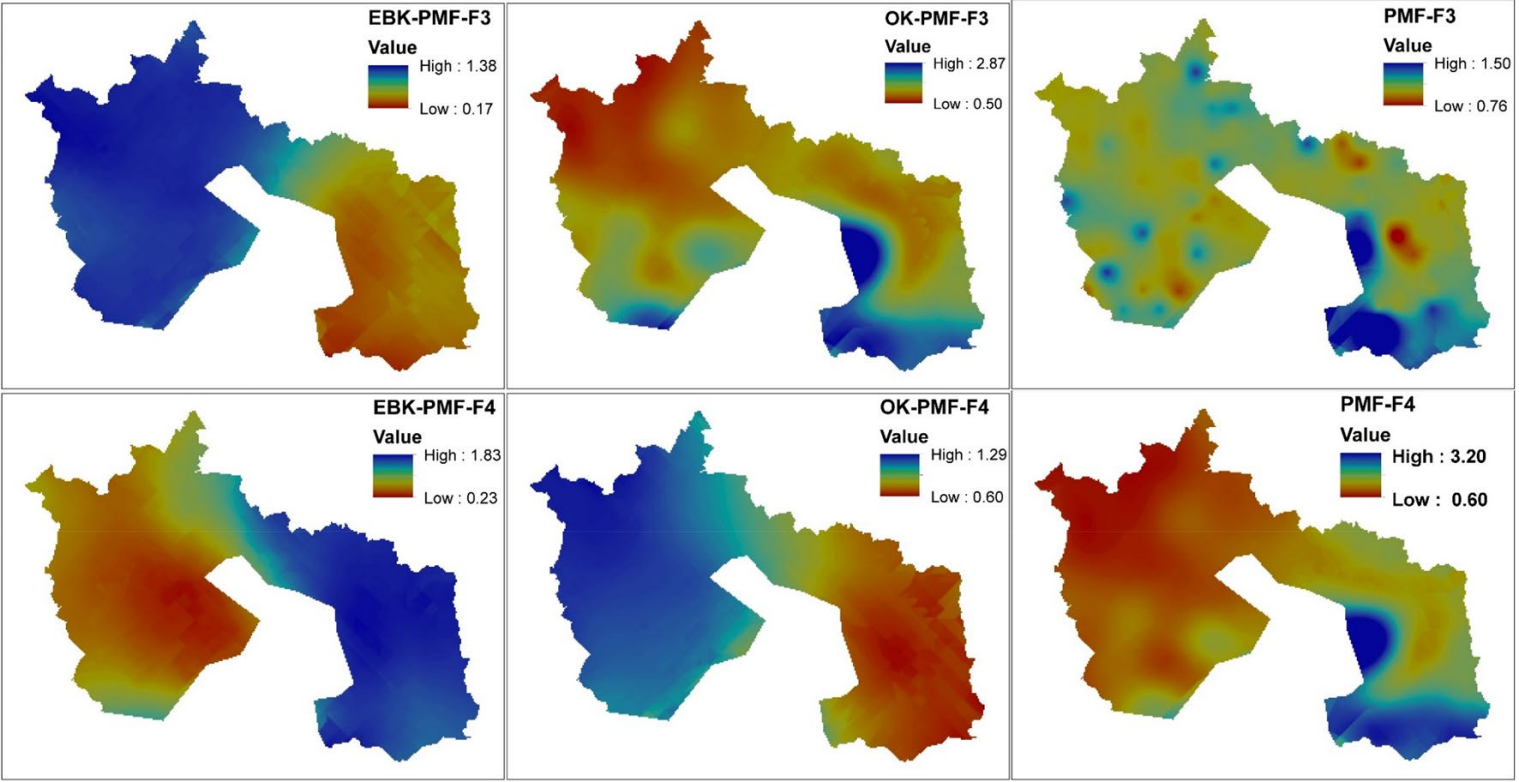
Spatial prediction of receptor model factor scores using geographically weighted regression kriging [The spatial distribution maps was created with ArcGIS Desktop (ESRI, Inc, Version 10.7, URL: https://desktop.arcgis.com)].

**Figure 5:**
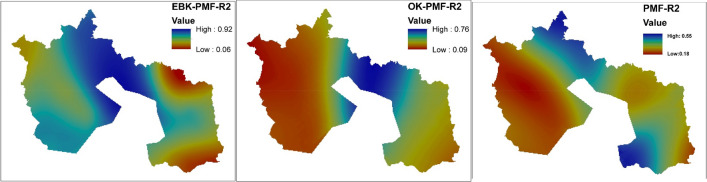
Spatial prediction of receptor model factor scores coefficient of determination (R^2^) using geographically weighted regression kriging [The spatial distribution maps was created with ArcGIS Desktop (ESRI, Inc, Version 10.7, URL: https://desktop.arcgis.com)].

The EPA-PMF version 5.0 was the software used to detect the source and compute the percentage contribution of each PTEs in each factor loadings. The accuracy of the analysis was guaranteed based on the minimum Q that controls the residual values E. The analytical process had to run 20 times to choose the best run that best fits data processed with a minimal Q value. Run 12 was deemed appropriate for this study, and four factors' loadings were discharged when all the runs converged (i.e., is signaling Yes). For a PTE to be deemed to have controlled a factor, the minimum percentage figure was fixed at 40%. Table 2 and Figs. 3, 4 and 5 indicate the percentage factorial contribution and spatial distribution of the geographical variation of the loaded PTEs in each factor per receptor model.

**Table 2:** Proportional contribution of each factor (F) for PTEs derived from receptor models.

	EBK-PMF				OK-PMF				PMF			
	F1%	F2%	F3%	F4%	F1%	F2%	F3%	F4%	F1%	F2%	F3%	F4%
Al	27.70	15.70	36.90	19.70	13.00	40.40	5.70	40.80	20.60	54.70	23.80	1.00
Ba	23.60	41.70	32.90	1.80	42.40	0.30	17.20	40.20	53.23	6.56	6.70	18.00
Cd	11.90	22.80	11.10	54.10	46.20	41.00	12.20	0.60	0.50	49.10	13.30	37.20
Fe	11.00	27.30	27.60	34.00	45.60	25.90	12.90	15.60	15.80	48.50	19.90	15.80
Pb	41.90	17.70	0.00	40.40	0.00	40.20	59.80	0.00	0.00	29.50	20.60	49.90
Sb	27.60	20.10	28.00	24.30	31.90	17.90	27.90	22.20	14.30	17.90	48.20	19.60
V	27.80	18.60	40.10	13.40	10.30	34.50	9.50	45.70	23.40	50.40	26.20	0.00

Factor 1 of the EBK-PMF receptor model accounted for 24.51% of the total variance in the factor loadings. On the other hand, OK-PMF and PMF receptor models accounted for 27.06% and 18.67% factor loadings, respectively. Pb (41.90%) dominated EBK-PMF, Cd (46.20%), Ba (42.40%), and Fe (45.60%) controlled OK-PMF, and Ba (53.23%) monopolized PMF (see Table 2). The distribution of PTEs in factor 1 of various receptor models suggested that Pb from the EBK-PMF and Cd from the OK-PMF are primarily anthropogenic in provenance. However, Ba from the PMF receptor model is more of a geogenic origin. Preceding studies by Ye et al.^62^; Ying et al.^63^ and Zhang et al.^64^ have suggested unequivocally that the excess of Pb and Cd in urban and peri-urban soil might be pollution emanating from vehicular traffic and other human activities such as particulate matter. According to a study conducted by Reitner and Thiel^65^, gasoline Pb additives were the principal source of Pb in the European atmosphere, which was deposited on the soil's surface. The authors also stated that Pb from road traffic is the primary contributor, with metallurgical production, immobile fuel combustion, and iron and steel fabrication playing a significant role. Phosphate fertilizers and waste incineration are two more primary sources of cadmium in the environment^66^. As a result, the prognosis for factor 1 of the geostatistical-based receptor models in the study area might be attributable to vehicular traffic and industrial sources (Pb), while Cd Phosphate fertilizers. Barium occurrence is mostly a geogenic source even though it does not exist in nature but in diverse forms such as barium sulphate and barium carbonates^67^. However, barium occurrence in the study area is more of a geogenic source, and this has been corroborated by the mean, maximum and minimum values quantified (see Table 1). Iron (Fe) is ubiquitous. Its concentration is mostly controlled by geogenic sources, which is consistent with a report on urban soil pollution in Bangkok by Wilcke et al.^54^ who claim that Fe concentrations appear to be controlled by the parent material. Although most literature suggests that Fe can be found virtually everywhere, its excesses in higher levels in soil and the environment may be traceable to a point source (e.g., iron and steel industries). Reitner and Thiel^65^, Alloway^57^ and Schafer and Einax^68^ hinted that the increased Fe concentrations in the environment and soil might be due to nearby industrial point sources producing iron-based substances such as iron and steel production, machine making, cast iron, wrought iron, and alloy as a significant source of Fe pollution in the environment. This correlates to the current situation in the study area, as evidenced by the presence of metal and steel industries.

Factor 2 of the EBK-PMF receptor model recorded a 23.42% variance in factor loadings, while the OK-PMF and PMF receptor models contributed 28.61% and 37.49%, respectively. Ba (41.70%) dominated factor 2 of the EBK-PMF receptor model, Al (46.20%), Cd (41.00%), and Pb (45.60%) controlled OK-PMF, and Al (54.70%), Cd (49.10%), Fe (48.50%), and V (50.40%) influenced PMF (see Table 2). PMF discharged a lot of dominant PTEs in this factor loadings more than the geostatistical based receptor models. However, the sources of Ba, Fe and Cd have been discussed previously in factor 1. Aluminum is ubiquitous and is mostly found in parent materials such as igneous rocks. According to Lantzy and Mackenzie^69^ and Exley^70^ Al is a significant component of the earth's crust; natural weathering processes go far beyond discharges to air, water, and land linked to human activity. According to Atsdr^71^, Al occurrence in the soil and the environment is via weathering rocks and minerals. However, the author further suggested that the man-made activities that pollute Al in the soil and environment are industrial processes, water effluent and atmospheric deposition. This is in line with the presence of the metal industry in the study area that produces aluminum products such as aluminum fences, aluminum sheets in all sizes, perforated sheets etc. Vanadium is distributed extensively in the igneous and sediment rocks and minerals^72^. Nevertheless, it is economically important because it is employed mostly in the steel sector in alloy manufacturing. Moskalyk et al.^73^ and Yu et al.^74^ outlined that vanadium reserves are discovered in mineral and hydrocarbon deposits worldwide, especially China, South Africa, and Russia, the biggest vanadium derivatives producers. The maximum vanadium value recorded is higher than the EAV threshold, implying that anthropogenic sources are augmenting the geogenic sources to elevate vanadium levels in certain areas of the study area near the steel plant.

Factor 3 of the EBK-PMF receptor model amassed 25.24% of the total variance in the factor loadings, whereas OK-PMF and PMF receptor models likewise accrued 20.75% and 23.18% of the total factor loadings, respectively. Factor 3 of the EBK-PMF receptor model was eclipsed by V (40.10%), OK-PMF was overshadowed by Pb (59.80%), and PMF was dictated by Sb (48.20%) (see Table 2). The sources of V and Pb in the study area have been discussed previously in factors 1 and 2. Antimony (Sb) is a hazardous PTEs that can be found in the environment. He^75^ outlined that many concerns have been raised about rising levels of Sb pollution in the environment, primarily because of anthropogenic activities and the widespread use of Sb compounds. When the measured concentration of Sb in the study area is compared to the WAV and EAV thresholds, it appears that the concentration is above the permissible limits. The high level of Sb in the environment and soil throughout the study area may be attributed to a variety of sources, including vehicular emissions for its use as a fire retardant in brake linings, waste disposal and incineration, fuel combustion, metal smelters, textiles, plastics, painting and coating industries. This is congruent with previous studies of Bradl^76^ and Tschan et al.^77^ who analyzed the origins and sources of PTEs in the soil and the environment.

Factor 4 of the EBK-PMF receptor model accounted for 26.83% of the total variance in the factor loadings. In contrast, OK-PMF and PMF receptor models likewise accumulated 23.60% and 20.67% of the total factor loadings, respectively. In factor 4, V (40.10%) controlled the EBK-PMF receptor model whilst OK-PMF was dominated by Al (40.80), Ba (40.20%) and V (45.70%), and PMF was dominated by Pb (49.90%) (see Table 2). The dominant PTEs have been discussed in the preceding factor loadings. Although Pb obtained a high percentage contribution from the OK-PMF, the receptor model consistently projected Pb as the dominant PTE in different factors such as factor 1 for EBK-PMF, factor 3 for OK-PMF and factor 4 for PMF receptor models.

The spatial distribution of the PTEs in each factor loadings was determined using geographical weighted regression kriging (see Figs. 3 and 4) on factor scores of each receptor model against four environmental covariates (i.e., elevation, total catchment area, LS factor, and valley depth), and the spatial prediction maps were duly evaluated for prediction accuracy using the coefficient of determination (R^2^). The receptor models displayed PTEs spatial distribution factor loadings hotspots for OK-PMF-F1 in the eastern area covering a more significant portion of the southeastern part of the map. Only EBK-PMF-F1 indicated patches of PTEs hotspots in the southeastern while both EBK-PMF- F1 and PMF-F1 hotspots were detected in the southwestern. OK-PMF-F2 and PMF-F2 maps shared similar patterns with PTEs distribution hotspots covering the northeastern to the southeastern part of the map. Nevertheless, the EBK-PMF-F2 map exhibited hotspots of PTEs in the southeastern sector of the map. The factor 3 maps of the receptor models also depicted massive spatial distribution hotspots for PTEs in the northwestern to the southwestern sector of the map for EBK-PMF-F3. However, the OK-PMF-F3 and PMF-F3 maps displayed patches of hotspots for the PTEs in factor 3. Factor 4 spatial distribution maps indicated PTEs pollution in the northeastern to the southeastern map area for EBK-PMF-F4. In the opposite direction, PTEs pollution hotspots were displayed for the OK-PMF-F4 map. Nonetheless, PMF-F4 showed a patch of hotspots on the southeastern side of the map.

The R^2^ distribution maps for the receptor models displayed similar hotspots patterns for EBK-PMF-R^2^ and OK-PMF-R^2^ (see Fig. 5) and on the contrary PMF-R^2^ map exhibited hotspots in the northwestern and the southeastern part of the map. The mapping prediction efficiency of the factor scores of the receptor models suggested that the EBK-PMF receptor model R^2^ values were between 0.05 and 0.92, whereas OK-PMF was between 0.09 and 0.76 and PMF was 0.18 and 0.55. This indicated that the prediction efficiency of the EBK-PMF receptor model efficiency factor scores was up to 92% as against 76% for OK-PMF and 55% for PMF receptor models, respectively.

### Reasonability and reliability of the results

Table 3 shows the source contribution results of the receptor models. Despite the fact that the source contributions to each factor loading came from a variety of sources, the source contributions in the table are based on the most prevalent PTEs and their dominance in factor loading per receptor model. Furthermore, while the source contribution per receptor model may be similar, it was distributed across a wide range of factor loadings. Therefore, the computed source contribution per factor loadings are reasonable and dependable, and diverse sources that contributed to quantifying the percentage proportion of PTEs pollution may be identified and interpreted. The correlation coefficient (R^2^), root mean square error (RMSE) and mean absolute error (MAE) of the performance of EBK-PMF/OK-PMF/PMF receptor models allocated by the algorithms indicates the reasonability and feasibility of the discovered source profiles or contributions in each factor loadings.

**Table 3:** Results from the different receptor models source contribution in each factor loadings.

Sources	EBK-PMF				OK-PMF				PMF			
	F1%	F2%	F3%	F4%	F1%	F2%	F3%	F3%	F1%	F2%	F3%	F4%
Geogenic	16.15	9.58	20.89	10.50	6.86	20.18	3.93	24.71	16.12	4.35	15.00	0.71
Vehicular traffic	13.76	25.44	18.63	0.96	22.39	0.15	11.85	24.35	41.64	0.52	4.22	12.72
Phosphate fertilizer	6.94	13.91	6.29	28.82	24.39	20.48	8.40	0.36	0.39	3.91	8.38	26.29
Steel industry	6.41	16.66	15.63	18.11	24.08	12.94	8.88	9.45	12.36	3.86	12.54	11.17
Atmospheric deposits	24.43	10.80	0.00	21.52	0.00	20.08	41.18	0.00	0.00	2.35	12.98	35.27
Metal works	16.09	12.26	15.86	12.95	16.84	8.94	19.21	13.45	11.19	1.42	30.37	13.85
Waste disposal	16.21	11.35	22.71	7.14	5.44	17.23	6.54	27.68	18.31	4.01	16.51	0.00

### Model performance

The performance of the receptor models was evaluated using the support vector machine regression (SVMR) and multiple linear regression (MLR) algorithms (see Table 4). The validation and accuracy evaluation criterion used results demonstrated that the R^2^ of both algorithms (SVMR and MLR) for the receptor models indicated that 5 of the 7 PTEs (Al, Ba, Pb, Sb and V) had high R^2^ values ranging from 0.915 to 0.996 for SVMR and 0.870 to 0.998 for MLR (Al, Ba, Pb and V). Thus, the EBK-PMF receptor model consistently had high goodness fit in 4 out of 7 PTEs for both algorithms applied. Furthermore, in both algorithms, four of the PTEs (Al, Ba, Pb and V) were consistently predicted to favor EBK-PMF receptor model. The marginal errors estimated for receptor models using the RMSE and MAE similarly suggested that the errors for EBK-PMF were significantly reduced for Al, Ba, Pb, Sb, and V employing the SVMR algorithm for both MAE and RMSE. The error was also considerably reduced for these PTEs (Al, Ba, Pb, and V) for the EK-PMF receptor model using the MLR algorithm. Similarly, Al, Ba, Pb, and V had lower error levels in both algorithms consistently for EBK-PMF compared to PMF and OK-PMF. The high R^2^ values and low error levels were anticipated based on comparable results achieved by Wu et al.^23^ when comparing APCS-MLR and PMF receptor models. In comparison, the minimum R^2^ value reported by Wu et al.^23^ was 0.83, whereas the minimum R^2^ reported in this current study is 0.87. Callén et al.^78^ comparative analyses in Spain suggested that PMF is optimal to UNMIX and APCS-MLR by comparing the computed R^2^ values and the marginal error of the PTEs when analyzed.

**Table 4:** Assessment of receptor models via support vector machine regression (SVMR) and multiple linear regression (MLR).

Algorithm	Models	Al			Ba			Cd			Fe		
		R^2^	RMSE	MAE	R^2^	RMSE	MAE	R^2^	RMSE	MAE	R^2^	RMSE	MAE
SVMR	EBK-PMF	0.968	0.113	0.083	0.996	0.043	0.036	0.981	0.092	0.071	0.978	0.097	0.072
	OK-PMF	0.758	0.286	0.158	0.932	0.157	0.085	0.994	0.047	0.037	0.988	0.064	0.052
	PMF	0.947	0.188	0.133	0.999	0.046	0.038	0.9	0.252	0.134	0.767	0.399	0.297

		Pb			Sb			V					
		R^2^	RMSE	MAE	R^2^	RMSE	MAE	R^2^	RMSE	MAE			
SVMR	EBK-PMF	0.993	0.06	0.045	0.915	0.19	0.141	0.98	0.092	0.07			
	OK-PMF	0.937	0.158	0.092	0.816	0.256	0.168	0.751	0.289	0.15			
	PMF	0.846	0.323	0.195	0.931	0.21	0.161	0.957	0.166	0.103			

		Al			Ba			Cd			Fe		
		R^2^	RMSE	MAE	R^2^	RMSE	MAE	R^2^	RMSE	MAE	R^2^	RMSE	MAE
MLR	EBK-PMF	0.873	565.369	464.727	0.993	1.131	0.899	0.995	0.05	0.039	0.986	573.108	463.86
	OK-PMF	0.939	895.006	670.332	0.843	4.289	3.416	0.997	0.037	0.029	0.986	506.787	432.985
	PMF	0.888	1215.43	932.4	0.998	1.713	0.98	0.946	0.228	0.165	0.891	3324.77	2471.66

		Pb			Sb			V					
		R^2^	RMSE	MAE	R^2^	RMSE	MAE	R^2^	RMSE	MAE			
MLR	EBK-PMF	0.998	0.472	0.352	0.674	0.259	0.189	0.87	0.752	0.605			
	OK-PMF	0.992	1.479	1.01	0.78	0.353	0.266	0.869	3.612	2.092			
	PMF	0.959	3.357	2.344	0.975	0.151	0.105	0.86	3.754	2.204			

Moreover, the authors^78^ added that the increased input data requirements of PMF enabled better results to be produced than with the other two models. This is congruent with the results of this study since the raw data was interpolated for EBK-PMF and OK-PMF, in which the predicted data extracted for the source apportionment computation improved modelling efficiency whilst significantly lowering errors in source distribution computation. Similarly, Gholizadeh et al.^20^ concluded that the APCS–MLR model performed better than the PMF due to its prediction efficiency based on R^2^ measured values. The cumulative performance of the hybridized receptor models in this study compared to the parent model (PMF) suggested that while the receptor models discharged relatively high R^2^ values, the error accompanying each source apportioned to each PTE in Ok-PMF and PMF is higher than EBK-PMF in terms of algorithms used. This is consistent with similar results obtained by Callén et al.^78^, reporting that the R^2^ was quite good, the errors, which were always in excess, were quite significant. Thus, the high errors in the receptor models could have impacted the output of the model (e.g., uncertainty parameters) and the data quality. Conversely, Gupta et al.^79^ compared different kriging interpolation algorithms and concluded that EBK interpolation enhances efficiency and, at the same time, reduces errors.

Most soils, particularly urban soils, exhibit pollution, compaction, and soil sealing, as well as deposition and the removal or mixing of natural substrates^80^. According to Bullock and Gregory^81^, soil throughout the urban and peri-urban setting appears to be highly impacted by human influence and even anthropogenic activities (i.e. carried from different places). A diversity of anthropogenic activities metes out these impacts. For instance, the urban and the peri-urban environment has been heavily influenced by vehicular emissions, coal burning, demolition or refurbishing of buildings, disposal of waste, metallurgy and urban paint usage^82^. These expose humans to all kinds of health-related challenges, especially children come into contact with PTEs related substances that are taken through diverse pathways such as dermal, ingestion and inhaling. Agyeman et al.^83^ reported that children exposed to PTEs in the urban and the peri-urban environment are higher due to their mouth and finger practices. The distinctive physiological of the youngsters, the hypersensitivity of the growing vital organ and various chemical types of metal is further exacerbated by the toxicological consequences^84^. The robustness of a receptor model with high efficiency and minimal error computation level tends to expose the hotspots of sources of PTEs in the environment and apportion in percentagewise the contribution of PTEs. The hybridization of EBK to PMF has achieved a high level of efficiency and minimize error significantly. This study demonstrated the viability of using a hybridized geostatistical-based receptor model to locate and distribute PTE sources in urban and peri-urban soils by applying and validating the EBK-PMF receptor model.

## Conclusion

One of the most efficient multivariate applications used to recognize the source pathways and apportion percentage contribution of PTEs in pollution-related determination is the application of receptor models. The study compared a parent receptor model PMF to hybridized geostatistical based receptor model OK-PMF and the EBK-PMF. The OK-PMF discharged more PTEs in each factor than the EBK-PMF and the PMF receptor model, respectively. Despite that, all the receptor models predicted PTEs distribution and identified respective sources in the study precisely and consistently. However, the validation and accuracy assessment computed using the R^2^, RMSE and the MAE via support vector machine regression and the multiple linear regression algorithms suggested that EBK-PMF was optimal for 5 out of the 7 PTEs analyzed using SVMR and 4 PTEs using MLR algorithms. Moreover, the errors estimated, and the prediction's efficiency also indicated that the EBK-PMF receptor model reduces that error margin significantly compared to the parent receptor model PMF and OK-PMF. In another vein, the GWRK spatial distribution map coefficient of determination prediction efficiency computed also suggested that the EBK-PMF receptor models factor scores prediction efficiency is up to 92% as against 76% for OK-PMF and 55% for the parent receptor model PMF. Therefore, this study recommends applying hybridized receptor model EBK-PMF in identifying the source pathways of PTEs and apportioning the percentage contribution of PTEs in a polluted environment.
